# Simulation of COVID-19 spread through family feast gatherings in a complex network

**DOI:** 10.1017/S0950268822000292

**Published:** 2022-03-02

**Authors:** Zuiyuan Guo, Lili Gong, Guangquan Xiao, Yayu Wang, Zhiwei Xu, Dan Xiao

**Affiliations:** 1 Department of Infectious Disease Prevention and Control, Center for Disease Control and Prevention in Northern Theatre Command, Shenyang, China; 2 Department of Psychiatry, General Hospital of Northern Theater Command, Shenyang, China; 3 China National Clinical Research Center for Neurological Diseases, Beijing Tiantan Hospital, Beijing, China

**Keywords:** COVID-19, dynamic model, feast, social contact network

## Abstract

Family feasting during the Spring Festival is a Chinese tradition. However, close contact during this period is likely to promote the spread of coronavirus disease 2019 (COVID-19). This study developed a dynamic infectious disease model in which the feast gatherings of families were considered the sole mode of transmission. The model simulates COVID-19 transmission via family feast gatherings through a social contact network. First, a kinship-based, virtual social contact network was constructed, with nodes representing families and connections representing kinships. Families in kinship with each other comprised of the largest globally coupled network, also known as a clique, in which a feast gathering was generated by randomly selecting two or more families willing to gather. The social contact network in the model comprised of 215 cliques formed among 608 families with 1517 family members. The modelling results indicated that when there is only one patient on day 0, the number of new infections will reach a peak on day 29, and almost all families and their members in the social contact network will be infected by day 60. This study demonstrated that COVID-19 can spread rapidly through continuous feast gatherings through social contact networks and that the disease will run rampant throughout the network.

## Introduction

The 7 days at the beginning of each year is the Spring Festival which is the most important traditional festival in China. However, in December 2019, the coronavirus disease 2019 (COVID-19) pandemic occurred in Wuhan, Hubei province, China, followed by the rapid spread to other cities in Hubei province and nationwide [1]. During the Spring Festival of 2020 and the subsequent 2 months, a series of strict nationwide control measures led to effective containment of the pandemic by the end of March 2020 [2–4]. Without these control measures during the 2020 Spring Festival, the pandemic would likely have evolved into an uncontrollable situation because of traditional family feast gatherings taking place instead of home-based quarantine.

Several COVID-19 infection events caused by feast gatherings in China occurred in many provinces during the Spring Festival in 2020, with some feast gatherings considered super-spreader events [5–7]. This indicated that feast gatherings obviously promote the spread of severe acute respiratory syndrome coronavirus 2. Though many related outbreaks have been reported, there are few references in the literature that use theoretical research to establish mathematical community spread models. Questions arose regarding whether continuous feast gatherings throughout society would lead to a marked spread of the pandemic and what situation would have arisen if feast gatherings had been allowed during the 2020 Spring Festival. Therefore, a dynamic infectious disease model is needed to gain deeper insights regarding these issues.

To date, many COVID-19 studies based on mathematical models of infectious disease have been conducted [8–15]. We previously established two individual-based computer models to analyse the impact of Wuhan's lockdown and that of asymptomatic-infected individuals on the pandemic's spread [16, 17]. Additionally, many studies have shown the value of complex network-based tools to simulate and analyse the spread of disease [18–21]. These pioneering interdisciplinary studies have contributed to the development of theoretical epidemiology. However, both the differential equation-based dynamic models and the individual-based computer models are underpinned by the traditional susceptible-exposed-infectious-recovered model, which assumes that viruses are transmitted through daily contact between susceptible and infectious individuals. However, although such events are an important and special means of disease transmission, few studies of family feast gatherings have been conducted.

Pandemic spread caused by family feast gatherings has some defining characteristics. First, susceptible and infected individuals are present simultaneously in the same small space; therefore, infections occur during the dining and gathering and only among the gathered participants. Second, the pandemic spreads among relatives in a social contact network rather than randomly among the whole population. Third, families at feast gatherings are familiar with each other; therefore, the nodes represented by the families are connected to each other.

Based on the above analysis, in this study, we proposed a model that simulates the spread of COVID-19 as a new and unknown infection, assuming that the early transmission stage occurred during the Spring Festival disease, based on family feast gatherings as a prevalent occurrence during this occasion. We considered the characteristics of such family feast gatherings in real social contact networks. First, we constructed a kinship-based, virtual social contact network with Chinese characteristics. Nodes in the social contact network represent families, and connections represent kinships. All the cliques are then identified in the social contact network. A clique is defined as the largest globally coupled network that cannot be extended by adding even one more node. Therefore, each clique represents a collection of families that have kinships. After searching all the cliques, we start to randomly identify the families participating in the feast on that day in each clique in turn, because on each day, there is a probability that any given family will decide to have a feast gathering. The epidemic will spread during the continuous feast.

We integrated a complex social contact network with the theory of infectious disease dynamics and incorporated Chinese behavioural habits into the model. This not only contributes to the existing research methodology but also ensures that the research findings are of high practical value. This proposed dynamic model of infectious disease represents a novel, non-traditional exploration of this research field.

## Methods

### Framework of the model

**Fig. 1.**
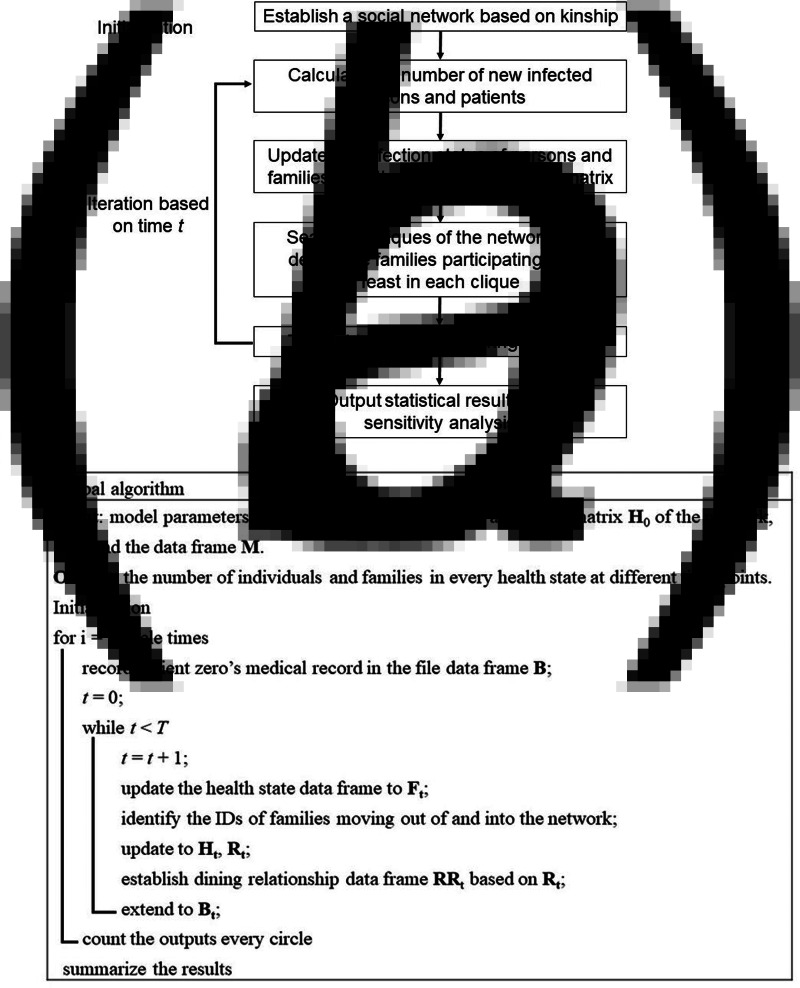
Flow chart of model design: model design framework (a) and global algorithm (b).

The framework of this model is as follows. First, we establish a virtual social network according to the kinship between families. Then, we start the following day iteration. We calculate the new infected individuals, new patients, new hospitalised patients and new convalescents on day *d*, and update the status of families and individuals. Furthermore, we search for the families that move out and those that move back into the network, and then we update the adjacent matrix of the network. Moreover, we find all the cliques in the network based on the updated adjacent matrix using the clique filtering algorithm and select the families participating in the feast in each clique according to the family's willingness to have the feast on day *d*. Then, we randomly determine the individuals who will be infected during the feast according to the infection status of each feast participant. The above iteration will continue until the specified deadline. Finally, the time distribution of the population is analysed. Specific steps are demonstrated in Figure 1a.

### Premises of the model

In order to make the model more accurate as well as concise, we adopted the following premises. First, the virus spreads only among individuals in kinships who attend feast gatherings and ignores transmission through daily contact. Second, natural births and deaths in the population are ignored, and the social contact network is considered a closed network without inflow or outflow of individuals. Third, all individuals are considered susceptible to the virus, although they cannot be infected again after they have been infected the first time. Fourth, feast gatherings occur only within a clique, that is, all families in a gathering are acquainted with each other. Fifth, the health status of each patient evolves through five periods: susceptibility, incubation, contagion, hospitalisation and discharge period (recovery or death). Finally, each family is allowed to have a maximum of one dining gathering each day.

### Construction of a social contact network

It is assumed that there are many families in a closed community. Each family is randomly allocated a certain number of members (from 1 to 4) based on the average family size in China [22]. Families that are related to each other by direct kinships comprise a clique that may consist of families including grandparents, uncles, aunts and father and mother, as well as families of their independent adult children.

Each family falls into one of two categories according to the marriage relationship: (1) there is a marital relationship. The married spouses live together or separately due to working in different places (they may live with their minor children or adult unmarried children). (2) There is no marriage. This relationship includes divorced persons, or unmarried single persons. The relatives of the former family type comprise husband-side relatives and wife-side relatives, which is the common node of the two cliques, thereby leading to at least a husband-side clique and a wife-side clique within the social contact network. In contrast, the relatives of the latter family type all belong to one clique. Moreover, it is assumed that 80% of families in a clique belong to the former family type and 20% of families in a clique belong to the latter family type.

Based on the above analysis, we began to establish a social network with family as the node and kinship as the connection. First, we set the total number of families in the community as 1000, and number these families from 1 to 1000. Then, according to the numbering order, we randomly select several families to form cliques, and the number of families in each clique is randomly selected from three to eight according to a uniform distribution. Based on the above operations, a data frame *E* can be constructed. Each row of *E* represents a clique, and the elements in each row represent the family number in the clique. At this time, there is no common node (family) in each clique. In order to connect these cliques to form a network, we need to build common nodes in the cliques. Since we have set that 80% of families in each clique form a common node with families in other cliques, we randomly select families in each clique according to this proportion as the common node. In this way, a symmetrical adjacent matrix *S* with elements only 1 and 0 can be constructed. Each row or column of *S* represents a clique, and element 1 (0) represents that two different cliques have at least 1 (0) common node. At the same time, we record the number of common nodes in each clique. Then, we successively replace the common nodes of each clique in *E* with the same number (the number of elements in *E* is <1000). Based on the updated E, an adjacent matrix *H* with only 1 or 0 elements can be established. Each row or column of *H* shows a family, and 1 (0) indicates whether the two families are relatives.

Moreover, in China, the husband's parents and wife's parents usually have gatherings with the couple. Accordingly, based on matrices *F* and *E*, we search the common nodes of all cliques in the network, and then select a portion of nodes that can have a feast with husband's parents and wife's parents with a probability of 0.5 (because not all husband's parents, wife's parents and the couple have a feast, we assume that the proportion of having a feast is 0.5). If it is determined that one family can establish a feast with the husband's parents and the wife's parents, then respectively randomly select one node as the parent family from the two different cliques where the family is located. Therefore, the three families also form a clique and mark it in *H*. For more details of the construction method of the network, refer to Appendix I.

### Identification of all cliques

All cliques in the network are identified using the clique filtering algorithm proposed by Palla *et al*. in 2005, and the family numbers that make up each clique are recorded [23]. Specifically, a clique search is performed for each node on the basis of *H*. The specific algorithm is as follows: when searching the clique containing the *m*-th node in *H*, translate the *m*-th row to the first row of *H*. Then, adjust the order of the columns so that element 1 in this row is concentrated on the left. Additionally, translate the second, third and so on rows of *H* in turn according to the above method. In this way, a maximum square matrix, which is the largest global coupling network (clique) of the *m*-th node, with all non-diagonal elements of 1 and all diagonal elements of 0 can be formed at the upper left of *H*. Since the rows are translated and the columns are translated accordingly, the translated matrix *H* is still a symmetrical matrix. Using this algorithm, we can identify all cliques that contain the given node. The serial numbers of the identified cliques and their families are stored in a data frame *R*. Each row of *R* represents a clique, and the elements of each row represent the families within that clique. Finally, duplicate cliques and their subsets in *R* are deleted (see Appendix II for more details on the algorithm).

Each family has a probability *P*, each day, of deciding to have a feast gathering. When deciding to have a feast gathering, the first step for the family is to find all cliques in *R* that contain the family. The second step is the random selection of a clique. Under normal circumstances, a feast gathering is considered to exist when at least two families in a clique choose to gather. However, when the parents-in-law and their children decide to have a feast gathering, three matched families must be involved at the same time. If only two families are matched, then the feast gathering is considered unrealisable. Eventually, all families that have decided to have a feast gathering but have failed to match are allowed to select a second clique for the planned feast gathering, although they will be excluded from consideration if that matched feast gathering fails to happen. Accordingly, families that have successfully engaged in feast gatherings in a clique comprise a subset of the clique. A data frame called *RR* is generated each day to record all successful feast gatherings in the social contact network, with each row representing a feast gathering and each element in the row representing the families involved in the gathering (see Appendix III for more details on the algorithm).

### Spread of the virus through feast gatherings

**Fig. 2.**
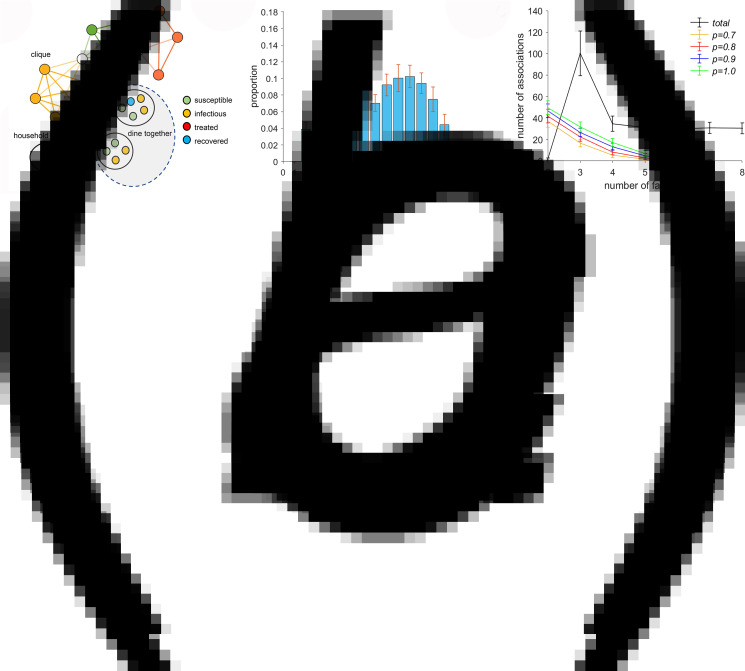
Static characteristics and clique structure of the social contact network. Local structure of the social contact network (a). Nodes represent families, and line connections represent kinships. The globally coupled networks in orange, green and red comprise 6, 5 and 4-family cliques, respectively. White nodes represent common nodes between two different cliques, and the black line links the husband's parental family to the wife's parental family. Each family has members with different health status. Distribution of the mean node degree and its 95% confidence interval (b). The number of *k*-family cliques (*k* = 2, 3, …, 8) in the social contact network (black line) and number of feast gatherings involving *n* (*n* = 2, 3, …, 7) matched families (other colour lines, each corresponding to a different value of probability *P*) (c).

**Fig. 3.**
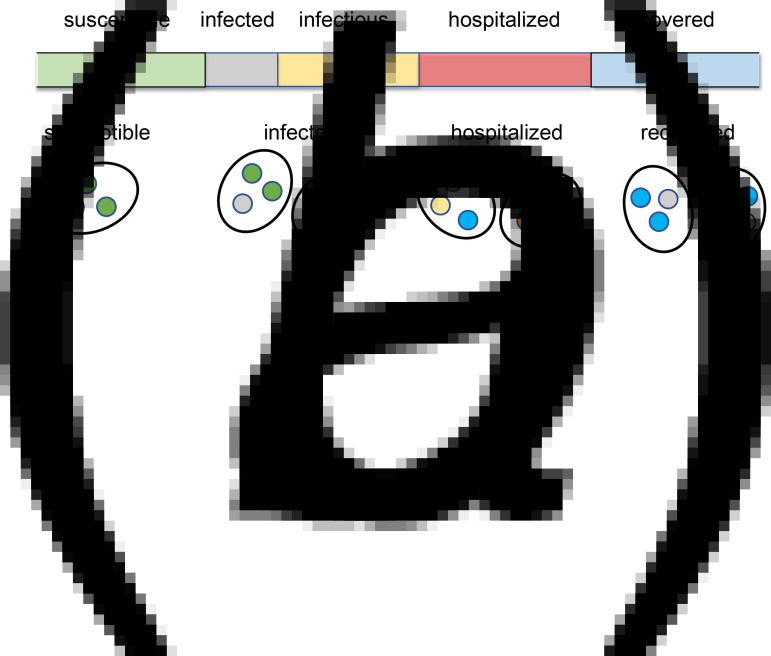
Health status of individuals and their families. Health status of individuals (a) and health status of families (b) are shown. In (a), infected indicates the incubation period. Infectious indicates the period from onset to hospitalisation. In (b), a susceptible family is a family in which all members are susceptible. An infected family is a family in which at least one member is infected, and no one is receiving inpatient treatment. A hospitalised family is a family in which at least one member is receiving inpatient treatment. Hospitalised families are removed from the network. A recovered family is a family in which all hospitalised members have been cured and discharged and have a willingness to re-attend feast gatherings.

It is necessary to update the data frame *RR* each day because the involvement of families in feast gatherings varies daily. All members of the matched families are involved in the gathering, which is conducted in the manner shown in Figure 2a. In a general scenario, there are infected individuals (infectors) and susceptible individuals attending the same feast gathering. During the feast, susceptible individuals become infected due to contact with infectors. Assuming that there are *m* infectors at a feast gathering and that there is a probability *q* for a susceptible individual to be infected by an infector at the feast gathering, it can be determined that there is a probability 1 − (1 − *q*)^*m*^ for a susceptible individual to be infected at the feast gathering. For a family that decides not to have a feast gathering on a given day or that plans to do so but fails to achieve it, the probability for a susceptible individual in the family to be infected on that day is calculated in the same way as described. A data frame *B* is generated to record the medical history of all infected individuals, with each row representing an infected individual and each column – from left to right – representing the serial number of the infection source, family serial number, personal serial number, infection time, onset time, hospitalisation time, discharge time and outcome (recovery or death). The health status of individuals and families is presented in Figure 3.

For a given individual, his or her participation in a feast gathering may be during the susceptibility period, incubation period, contagion period (from onset to admission) or discharge period after recovery. Patients do not attend feast gatherings when hospitalised or dead. The reason why some patients attend feast gatherings despite illness symptoms is that they mistakenly consider their symptoms to reflect a common respiratory disease and fail to give those symptoms sufficient attention. When a new infected person is generated, a new row is added below the last row of *B* to record the medical information of the new infected person. With the epidemic spread, the number of *B* rows is increasing. The parameters of the model are shown in Table 1.

**Table 1.** Model parameters

Description	Distribution characteristics	Numerical values	Sources (references)
Incubation period	Lognormal distribution	*μ* = 5.2, *σ* = 0.87	[1]
Infectious period (mean duration from onset to hospital admission estimated as 12.5 days, 95% CI, 10.3‒14.8 days)	Weibull distribution	Shape parameter, *m* = 1.66 Scale parameter, *η* = 8.73	[1]
Duration of hospitalisation	Uniform distribution	12‒20 days	[27]
COVID-19 case fatality rate	Bernoulli distribution	0.023	[28]
Average daily probability *p* that a family chooses to have a feast gathering	Bernoulli distribution	0.8	Assumed
Probability *q* that a susceptible individual becomes infected because of attending a feast gathering with one infector	Bernoulli distribution	0.8	Assumed
Distribution of Chinese family sizes	Bernoulli distribution	Probabilities that the family size is 1, 2, 3 and 4 are 0.2, 0.33, 0.28 and 0.19, respectively	[22]

CI, confidence interval; COVID-19, coronavirus disease.

Moreover, when at least one patient in the family is hospitalised or dies of illness, other members of the same family will no longer attend any feast gathering out of ethical considerations; therefore, the family is removed from the social contact network. When all patients in a family have been cured and discharged, the family returns to the social contact network and continues to attend feast gatherings. Because the health status of individuals and that of families in the network may change on a daily basis, it is necessary to generate a data frame *F* to record the health status, with each row representing a family, the first element of each row representing the status of the family (1 means ‘removed’, and 0 means ‘not removed’), and subsequent elements representing the health status of family members (numbers 1–6 represent the susceptibility period, incubation period, contagion period, treatment period, death and recovery, respectively). According to the family status recorded in the data frame *F*, it is possible to find the sequential numbers of families that are removed from and are added back into the social contact network each day while updating the adjacent matrix *H* and clique data frame *R*. Based on the personal status recorded in *F*, it is possible to find the susceptible individuals, recovered individuals and infectors attending the same feast gathering. When the model end time *T* is reached, the iteration ends. The infection time, onset time, hospitalisation time and discharge time of infected individuals, as well as the daily numbers of families with different status, are statistically analysed on the basis of *B*. The results are presented as a diagram showing the temporal distribution of the pandemic situation (see Fig. 1b and Appendix IV for more details on the global algorithm).

*S*, *E*, *I*, *H* and *R* represent the number of susceptible, exposed (incubation period), infectious, hospitalised and recovered individuals, respectively. Their variations on day *d* are expressed by the following formula:1
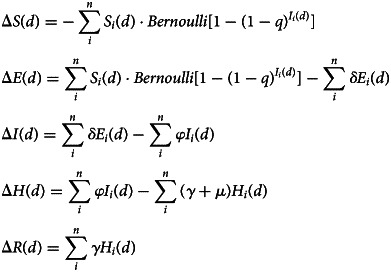


In formula (1), Δ*S*(*d*) = *S*(*d*) − *S*(*d* − 1) (1 ≤ *d* ≤ *T*), *i* represents the *i-*th feast, *n* represents the total number of feasts on day *d*, *δ* represents the probability that the infected person in the incubation period is transformed into a patient per day, *φ* represents the probability that the patient is transferred to the hospital per day, *γ* represents the probability that the hospitalised patient recovers per day and *μ* represents the probability that the hospitalised patient dies per day.

### Sensitivity analysis

Partial rank correlation coefficients (PRCCs) and Latin hypercube sampling (LHS) were used to conduct the sensitivity analyses. PRCC–LHS is an efficient and reliable sampling-based sensitivity analysis method that provides a measure of monotonicity between a set of parameters and the model output after removing the linear effects of all parameters except for the parameter of interest [24]. Each parameter interval was divided into *N* smaller and equal intervals, and one sample was selected randomly from each interval [24, 25]. A standard coefficient denoting the correlation between the parameter and model output was calculated. All analyses were conducted using MATLAB R2019a software (MathWorks, Natick, Massachusetts, USA).

## Results

### Network characteristics

A social contact network comprising 608 families with 1517 family members was constructed with a mean node degree of 8.45, mean network diameter of 8 and mean clustering coefficient of 0.63. The distribution of the node degree (Fig. 2b) showed that a node degree of 7 accounted for 14% of the total nodes, which was significantly higher than those accounted for by other node degrees. The network comprised of 215 cliques, of which three-family cliques were most frequent, followed by similar numbers of cliques each comprising four–eight families. If each family had a fixed probability, *P*, of deciding to attend a feast gathering, then the number of feast gatherings would gradually decline with increasing numbers (2, 3, …, 7) of matched families. However, the number of feast gatherings increases with increasing *P* – the willingness of a family to have a feast gathering (Fig. 2c).

### Changes in the pandemic situation

**Fig. 4.**
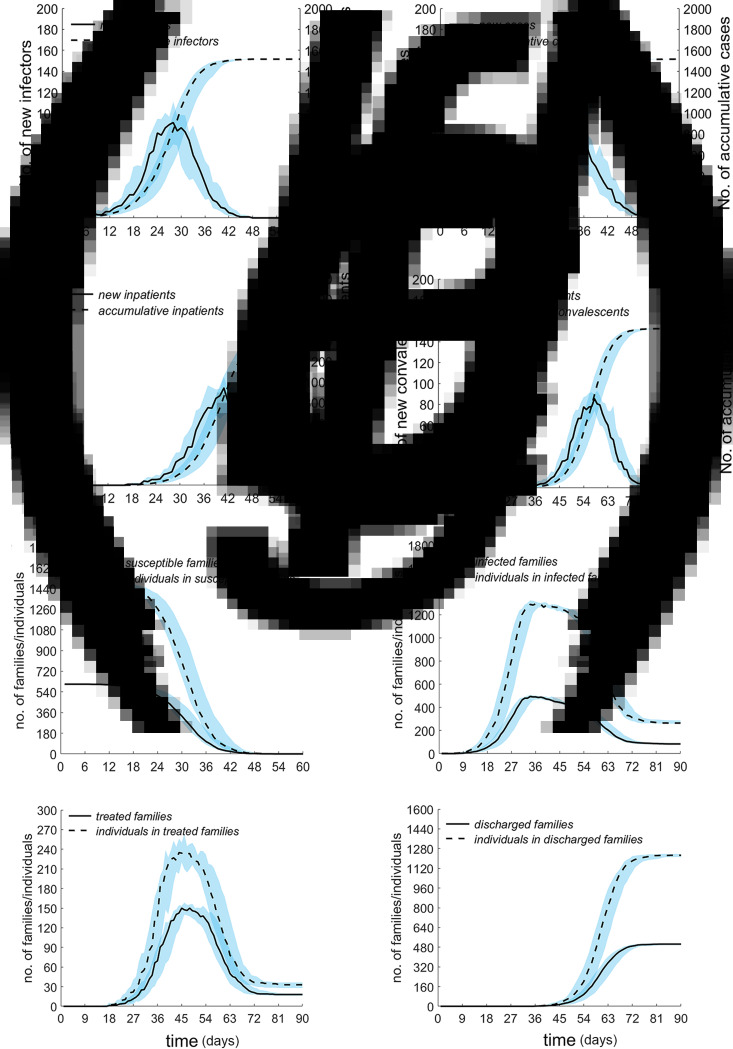
Temporal distribution of the pandemic situation. (a) Temporal distribution of the number of new infectors and cumulative number of infectors. (b) Temporal distribution of the number of new cases and cumulative number of cases. (c) Temporal distribution of the number of new inpatients and cumulative number of inpatients. (d) Temporal distribution of the number of new discharges and cumulative number of discharges. (e) Temporal distribution of the total numbers of susceptible families and their members. (f) Temporal distribution of the total numbers of infected families and their members. (g) Temporal distribution of the total numbers of hospitalised families and their members. (h) Temporal distribution of the total numbers of recovered families and their members. These results have a significance level of *P* = 0.8. The blue areas represent the 25‒75% percentiles of the values.

The changes in the number of infected individuals over time are shown in Figure 4a. The time spans were extended to 60 and 90 days to fully examine the evolution of the pandemic situation. The number of new infectors grew very slowly before day 12; however, from day 12, this number increased rapidly and reached the maximum of 91 (25‒75% percentile (50% P): 70‒111) on day 29, followed by a rapid decline. The cumulative number of infections reached 1515 on day 60. Additionally, the numbers of cases, inpatients and discharges (including the recovered and the dead) over time (Fig. 4b–d) all present a similar trend to the number of new infectors. The maximum numbers of new cases, new inpatients and new discharges reached 83 (50% P: 57‒90) on day 31, 94 (50% P: 68‒105) on day 42 and 85 (50% P: 74‒90) on day 59, respectively. The cumulative number of cases and inpatients reached 1515 and 1513, respectively, on day 60, whereas the cumulative number of discharges reached 1510 on day 90.

**Fig. 5.**
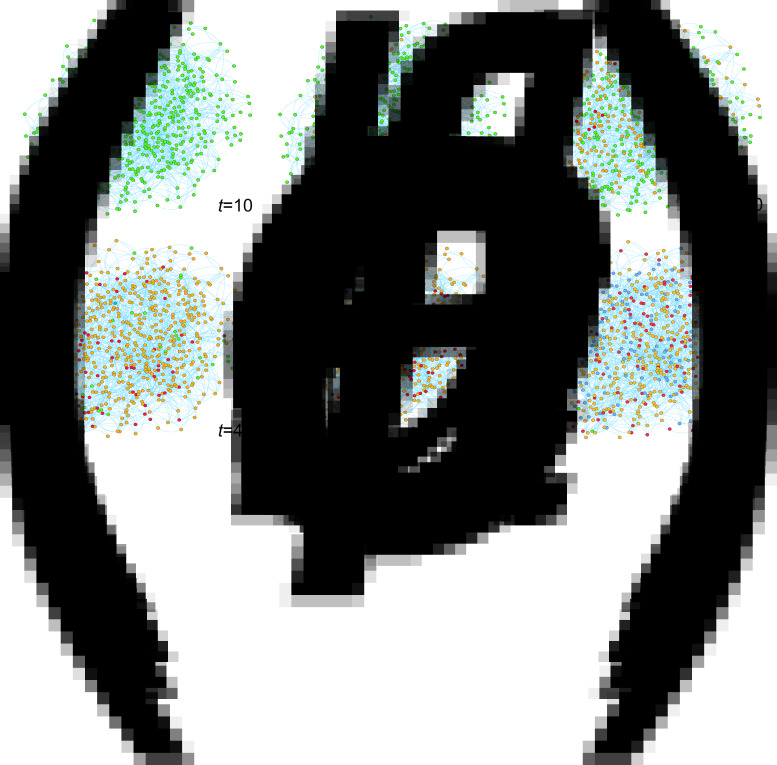
Temporal evolution of family status in the social contact network. The family status when (a) *t* = 10 days, (b) *t* = 20 days, (c) *t* = 30 days, (d) *t* = 40 days, (e) *t* = 50 days and (f) *t* = 60 days. Each node represents a family. Green represents susceptible families, orange represents infected families, red represents hospitalised families and blue represents recovered families.

The total numbers of susceptible families, infected families, hospitalised families and recovered families over time are shown (Fig. 4e–h). The total numbers of susceptible families and their members are 607 and 1512 on day 0, respectively. The total numbers of infected families and their members reached a maximum of 494 (50% P: 436‒508) on day 34 and a maximum of 1294 (50% P: 1241‒1314) on day 36, respectively. The total numbers of hospitalised families and their members reached a maximum of 150 (50% P: 126‒154) on day 45 and 235 (50% P: 203‒256) on day 44, respectively. On day 90, the total numbers of recovered families and their members reached 505 (50% P: 497‒511) and 1226 (50% P: 1212‒1237), respectively. The trends over time of all the families' status are shown in Figure 5. The median and fluctuation range of the above results are obtained through 100 time calculations.

### Basic reproduction number *R*_0_

*R*_0_ represents the number of individuals infected by an infector during the infection period in an environment where all individuals are susceptible, with *R*_0_ > 1 indicating a tendency for pandemic spread and *R*_0_ < 1 indicating a tendency for the pandemic ending. Assuming that during the infection period *τ*, only patient zero is contagious while other individuals who are sequentially infected at a later time are currently in the incubation period and are not contagious, *R*_0_ can be expressed as:2
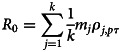


In equation (1), *k* represents the number of cliques that contain the family of the infector, 1/*k* represents the probability that the infector chooses clique *j* to attend a feast gathering; *m_j_* represents the number of individuals in clique *j*; *p* represents the probability that a given family chooses to have a feast gathering on a given day; *pτ* represents the number of feast gatherings attended by the infector during the infection period and *ρ*_*j*,*pτ*_ represents the infection rate of clique *j* after the infector has attended *pτ* feast gatherings. Accordingly, the following equations are applicable:3
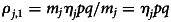
4
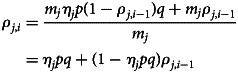
where *η*_*j*_ represents the probability that families willing to have a gathering choose to gather in the *j*-th clique and *q* represents the probability that a susceptible individual will be infected when coming into contact with an infector. According to equations (2) and (3), *ρ*_*j*,*pτ*_ = 1 − (1 − *η*_*j*_*pq*)^*pτ*^, if *ρ*_*j*,*pτ*_ is substituted into equation (1), let *η*_*j*_ = *η* (*j* = 1, 2, …, *k*), thereby allowing equation (1) to be re-written as:5
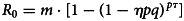


**Fig. 6.**
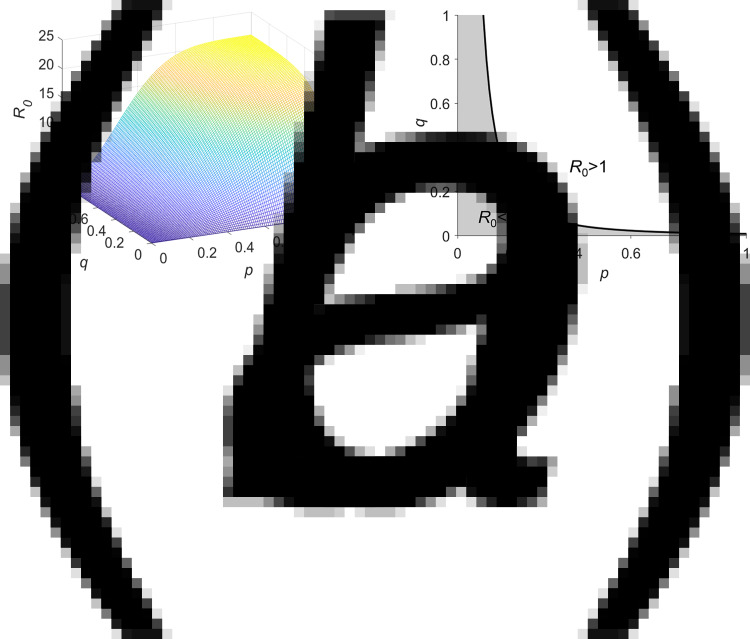
Distribution of the basic reproduction number. Relationship between the dependent variable *R*_0_ and independent variables *p* and *q* when *m* = 21, *η* = 0.5 and *τ* = 12 (a). The shaded part is generated by using the plane of *R*_0_ = 1 to cut the curved surface in (a) and projecting the lower remaining part on the *p*‒*q* plane (b).

In equation (4), 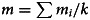 represents the mean number per clique, *p* and *q* are independent variables and *m*, *η* and *τ* are parameters. The relationship of *p*, *q* and *R*_0_ is presented in Figure 6a. When *R*_0_ = 1, the relationship between *p* and *q* represents the curve in Figure 6b. When *R*_0_ > 1, the pandemic will spread in the network; when *R*_0_ < 1, the pandemic will end.

### Sensitivity analyses

**Fig. 7.**
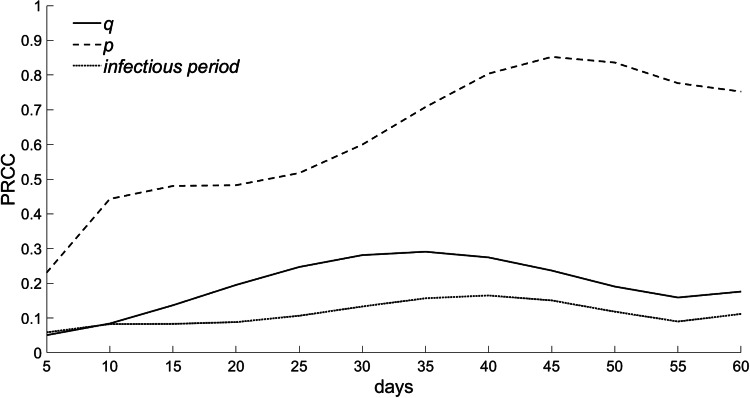
Sensitivity analysis of the continuous time series. *p* represents the average daily probability that a family chooses to have a feast gathering. *q* represents that the probability a susceptible individual becomes infected because of attending a feast gathering with one infector.

In this study, sensitivity analyses were conducted with three parameters (*p*, *q* and infectious period) and a continuous time series for the cumulative number of infected persons. We took *N* = 100 samples from a uniform distribution for each parameter range. PRCCs near 1 indicate that the parameter has a strong impact on the output, whereas those closer to 0 indicate less effect on the output result for that parameter (Fig. 7). The results reflected that the three parameters were positively correlated with the cumulative number of infected persons, which was consistent with the objective facts, and *p* had more effect than *q* and the infectious period. This result can be indirectly confirmed by the expression of *R*_0_ (formula (5)). Because the dependent variable 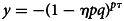 increases with the increase of the variables *p*, *q*, *τ* (*τ*, namely infectious period), and *R*_0_ = *m*(1 + *y*) is also an increasing function of *y*. As a result, *R*_0_ is positively correlated with *p*, *q* and *τ*, and the greater *R*_0_, the more infected people increase. In this formula, *p* appears more than *q* and *τ* once, so that the change in *p* has a greater impact on *R*_0_, which can explain that the PRCC of *p* is greater than *q* and *τ*. The above analysis confirms that the sensitivity analysis as a confirmation of the effectiveness and reliability of the model not only has scientific basis but also comply with the objective rules.

## Discussion

### Construction of a social contact network

**Fig. 8.**
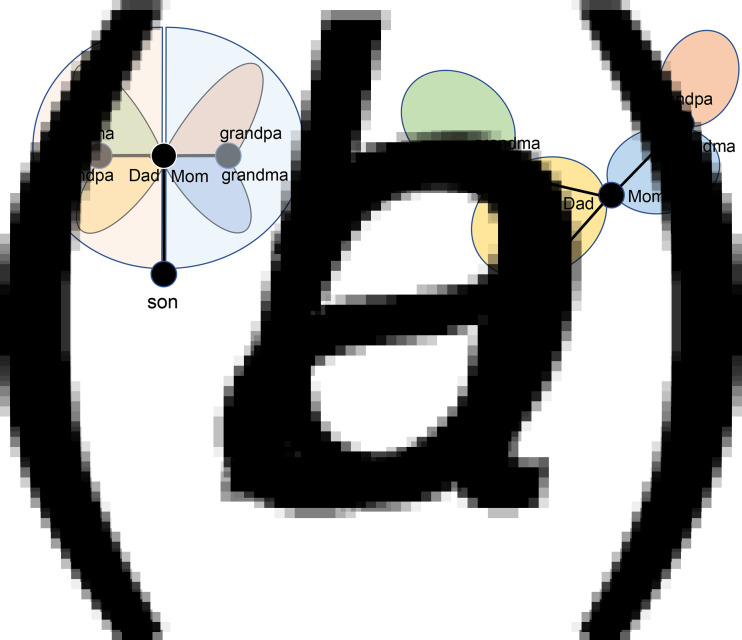
Kinship diagram. Adult married children belong to both the father's clique and mother's clique (a). Areas in different colours represent different cliques. Adult married children randomly belong to the father's clique or the mother's clique (b). Dad = father; grandma = grandmother; grandpa = grandfather; mom = mother.

One difference between family feast gatherings and social dining gatherings is that the former participants are kin and are acquainted with each other. The proposed model is supported by the assumption that the main members of a family are the espoused couple. Therefore, their relatives belong to two independent cliques, with either clique related to one spouse/partner or the other. Accordingly, the kinship-based cliques of an individual should include both paternal relatives (grandparents, uncles, aunts, cousins, nieces and nephews) and maternal relatives (grandparents, uncles, aunts, cousins, nieces and nephews) (Fig. 8a). However, when constructing cliques according to this method, it is necessary to specify the kinship among all families in each clique and to build a kinship database. Such practice not only complicates network construction, but also leads to large deviations of the constructed network from the real network. To circumvent this problem, we randomly allocated adult children's families to one of the two parental cliques (Fig. 8b), thereby greatly reducing the network construction complexity. In addition to this clique construction approach, we also consider the scenario that a family has a feast gathering with both the husband's parental family and wife's parental family, as is common practice in China. Moreover, the three-family feast gathering is regarded in this study as a joint gathering of the families of the husband's siblings and wife's siblings with the family of the spouses.

### Pandemic spread through family feast gatherings

We have previously developed a computer model to simulate the spread of type 7 adenovirus among soldiers through daily contact in a closed military camp [26]. That model revealed temporal trends similar to those of the present study in terms of the number of new infections and cumulative number of infections, suggesting that a general mechanism underlies infectious disease spread. The present study does not consider disease prevention and control measures or population immunisation because disease that spreads through continuous feast gatherings can only occur during the early stage of the social spread of an emerging infectious disease when the population is susceptible to the disease, such as in COVID-19, without awareness. It is conceivable that if the Chinese government had not implemented intervention measures in a timely manner, then the situation modelled here would have occurred in China during the 2020 Spring Festival. The present modelling results also show that when the parameters remain fixed, the magnitude of *R*_0_ in the model is determined by the two variables *p* and *q*. An intuitive explanation for this observation is that the higher the frequency of feast gatherings, the stronger the infectiousness of the disease and greater the *R*_0_.

### Limitations of this study

This study had four main limitations. First, the modelled social contact network is virtual, and the real kinship network can be known only through a large-scale census, which was beyond the technical scope of this study. Therefore, the real social contact network can be simplified to only a moderate extent, and such simplification introduces bias. Second, holiday feast gatherings in the real world can happen not only between relatives and family members, but also between friends, and they can sometimes involve strangers. Additionally, the virus can infect not only the participants of a feast gathering, but also other individuals, such as the staff as well as other guests, if the gathering occurs at a restaurant. However, only the simplest scenario was considered in this study to develop a model that is hypothetical yet easy to use. Third, the probability of a daily family feast gathering was set to 0.8 to make the model results more salient to highlight the main point of this study. However, this value is, in fact, lower under normal circumstances. Moreover, the modelled time span was extended to 60 days, although continuous involvement in feast gatherings during this period is unrealistic. Therefore, the present results overestimate the severity of pandemic spread promoted by feast gatherings. Fourth, due to the fact that epidemic data that describe the spread caused by continuous family feasts does not exist in reality, we cannot estimate the parameters and verify the effectiveness of our prototype by model fitting.

## Conclusions

The model appropriately simulated the dynamic spread of COVID-19 through family feast gatherings in a social contact network and provided a theoretical basis for the formulation and implementation of public health policies. Continuous feast gatherings can cause an increased spreading of disease, such as COVID-19, among the population; as do similar activities of high frequency and familial close contact occurrence, such as weddings, religious gatherings and even funeral services. Therefore, it is necessary to take measures, such as self-quarantine, social distancing and reducing crowd gatherings, during the early stage of such an outbreak to end the pandemic. This study further confirmed that during the epidemic spread, some public health decision-makings implemented by the government such as community control, online office and teaching, calling on residents to reduce social activities can effectively control the spread of the epidemic.

## Data Availability

The datasets generated or analysed during the current study are available in the Appendixes.

## Supplementary material

For supplementary material accompanying this paper visit https://doi.org/10.1017/S0950268822000292. click here to view supplementary material
