# Supplementary material for: Simulation of COVID-19 spread through family feast gatherings in a complex network
Source: Epidemiol Infect. 2022 Mar 2;150:e61. doi: 10.1017/S0950268822000292 (PMC8924592; doi:10.1017/S0950268822000292)
Supplement: Supplementary file 1 [file hygsup.zip › S0950268822000292sup004.pdf]

```
clear all
nfam=1000;                                        %Initial number of families
bn=8;sn=3;
Rf=[];                                            %Store the number of cliques
i=0;
while sum(Rf)~=nfam
        i=i+1;
        Rf=round(rand*(bn-sn+1)+(sn-0.5));   %Equalitily and randomly generate a clique of 3-8 families
        while sum(Rf)<nfam
                Rf=[Rf,round(rand*(bn-sn+1)+(sn-0.5))];
        end
end
E=[];                                             %Used to store all cliques and families
rnfam=1:nfam;
for i=1:length(Rf)
        er=rnfam(1:Rf(i));
        E(i,1:length(er))=er;
        rnfam(1:Rf(i))=[];
end
E2=E;

%%%Find the connection between cliques and store them in matrix F
sRf=round(Rf*0.8);                        %The number of nodes connected by each clique to other cliques
sRf0=sRf;
suRf=zeros(1,length(sRf));                %Used to store the sum of the degrees of the columns of F
Rf01=zeros(1,length(sRf));                %Used to determine whether the degree of each column is equal to sRF
F=zeros(length(sRf),length(sRf)); %Used to represent the connection matrix between cliques
k=0;
for i=1:length(Rf)-1
        if Rf01(i)==0
                lRf01=find(Rf01(i+1:length(Rf01))==0); %If the element in Rf01 is equal to 0, the maximum connectivity is not reached
                if sRf(i)<sum(F(i,1:i))+length(lRf01)
                        ran=randperm(length(lRf01),sRf(i)-sum(F(i,1:i))); %Find the order of nodes that can be selected
                        rm=lRf01(ran)+i;                        %Find the node that can be selected
                        suRf(rm)=suRf(rm)+1;                    %Update uRf
                        Rf01=sRf==suRf;                         %UpdateRf01
                        F(i,rm)=1;F(rm,i)=1;                    %UpdateF
                    else
                        Rf01(i)=1;
                        sRf(i)=sum(F(i,1:i));
                        k=k+1;
                end
        end
end
```

```
find(sRf0-sRf~=0);

%%%Establish the final social network, each node represents a family
G=[];                                                   %G is used to store nodes that need to be changed
for i=1:length(Rf)
        G(i,1:sRf(i))=E(i,randperm(Rf(i),sRf(i)));
end

%%Get the E after updating the same node
ran=ones(length(Rf),1);                                 %It is used to store the position that has been replaced
for i=1:length(Rf)-1
        ro=find(F(i,i+1:length(Rf))==1)+i;              %Indicates the line of the family in E that needs to be replaced
        for j=1:sRf(i)-ran(i)+1
                co=find(E(ro(j),:)==G(ro(j),ran(ro(j))));      %Co denotes the column to be replaced in E
                E(ro(j),co)=G(i,ran(i));
                ran(i)=ran(i)+1;
                ran(ro(j))=ran(ro(j))+1;
        end
end

%%Establish the final matrix H
%%Calculating the order of H
eh=[];                                  %The matrix E is transformed into row vector and stored in eh
for i=1:length(Rf)
         Er=E(i,:);
         eh=[eh,Er(find(Er>0))];
end
i=1;
while i<length(eh)
        eh(find(eh(i+1:length(eh))==eh(i))+i)=[];       %eh is a vector with non repeating elements
        i=i+1;
end

H=zeros(nfam,nfam);
for i=1:length(eh)
        [m,n]=find(E==eh(i));                   %eh represents the the row of H
        [x,y]=size(E(m,:));
        ec=reshape(E(m,:),1,x*y);
        ec(find(ec==0))=[]; ec(find(ec==eh(i)))=[];     %e0 represents the a column of H
        H(eh(i),ec)=1;H(ec,eh(i))=1;
end

%%Collecting the relatives by marriage
i=1;
```

```
while sum(F(i,i+1:length(Rf)))>0
        rc=find(F(i,i+1:length(Rf))>0)+i;          %fc denotes the clique connected to the i-th clique
        EL=E([i,rc],:);                             %El denotes all cliques connected to clique i
        su=sum(F(i,i+1:length(Rf))>0);              %su is the clique coefficient connected to the ith clique
        n=round((su-1)/2);
        ran=round(rand(1,n)*su+0.5);                %ran represents a row randomly selected from EL
        for j=1:n
                ELr=EL([1,ran(j)+1],:);             %ELR denotes the clique connected to the first clique in EL
                u=0;                                %Set the condition for finding two cliques with the same element
                k=0;                                %The position of the element in ELr1
                while u==0                          %Find the same elements in ELr
                        k=k+1;
                        ELr1=ELr(1,:);ELr2=ELr(2,:);
                        f=find(ELr2==ELr1(k));
                        u=sum(f);
                end
                r1=E(i,:); r2=E(rc(ran(j)),:);
                r1(find(r1==ELr1(k)))=[]; r1=r1(find(r1)>0);
                r2(find(r2==ELr2(f)))=[]; r2=r2(find(r2)>0);
                ran1=r1(round(rand*length(r1)+0.5));
                ran2=r2(round(rand*length(r2)+0.5));
                H(ran1,ran2)=1;H(ran2,ran1)=1;
        end
        i=i+1;
end
%Remove the unconnected nodes from the network
H(find(sum(H)==0),:)=[];
H(:,find(sum(H)==0))=[];
```
