# Supplementary material for: Simulation of COVID-19 spread through family feast gatherings in a complex network
Source: Epidemiol Infect. 2022 Mar 2;150:e61. doi: 10.1017/S0950268822000292 (PMC8924592; doi:10.1017/S0950268822000292)
Supplement: Supplementary file 1 [file hygsup.zip › S0950268822000292sup003.pdf]

```
clear all
A=H;
%%%Arrange the first row and first column first
sumStore=[];                                          %It is used to store the total number of cliques
Sum0=sum(A,2);
Nf=length(Sum0);
greater2=find(Sum0>0);                                %greater2 stores nodes with degree greater than 1

for bxh=1:length(greater2)                           %Each node with degree greater than 2 is regarded as the node of initial calculation, and Bxh is the number of cycles
        r=1:Nf;
        A=H;
        r1=greater2(bxh);              %r1 indicates the order of the currently moved rows
        Rdown=[];                      %It is used to store each rdown of each calculation node
        rdown=sum(A(r1,:))+1;          %Calculating the initial value of rdown
        Rdown=[rdown;Nf];              %Update Rdown
        Ar1=A(r1,:);                   %Ar1 represents the row currently moved
        Ar1(r1)=Ar1(1);Ar1(1)=0;       %Data exchange between two rows
        Ar0=A(1,:);
        Ar0(1)=Ar0(r1);Ar0(r1)=0;
        A(1,:)=Ar1;A(r1,:)=Ar0;
        A(:,1)=Ar1';A(:,r1)=Ar0';
        r(1)=r1;r(r1)=1;

        %%First line internal adjustment
        Ar=A(1,:);                     %Find the row to adjust
        Fr0=find(Ar==0);
        Fr1=find(Ar==1);
        if length(Fr0)>1
                for i=1:(length(Fr1)-(Fr0(2)-2))        %Start to adjust
                        h0=Fr0(2)-1+i;
                        Ar=A(1,:);
                        Find1=find(Ar((h0+1):Nf)==1);
                        h1=h0+Find1(1);
                        Artemp0=A(:,h0);                              %0 column
                        Artemp0(h0)=Artemp0(h1);Artemp0(h1)=0;
                        Artemp1=A(:,h1);                              %1column
                        Artemp1(h1)=Artemp1(h0);Artemp1(h0)=0;
                        A(:,h0)=Artemp1;                              %adjust the 0 column
                        A(:,h1)=Artemp0;                              %adjust the 1 column
                        A(h0,:)=Artemp1';                             %adjust the 0 row
                        A(h1,:)=Artemp0';                             %adjust the 1 row
                        r0=r(h0);r(h0)=r(h1);r(h1)=r0;
                end
        end
```

```
rup=2;                                                  %Upper bound of the number of rows calculated
while rup<Rdown(1)
        Fm=A(rup:Rdown(1),rup:Nf);                      %Fm is the matrix to be screened
        %%Choose the right line
        lFm=1:Rdown(1)-rup+1;                           %LFM is the number of rows of RFM
        m=1;
        while length(lFm)>1                     %If the number of rows LFm is greater than 1,
                                                %the number of columns of the matrix RFm is continuously expanded
                if m<length(Rdown)
                        RFm=Fm(lFm,1:Rdown(m)-rup);
                        %Update the extended Fm to RFm, and search from small to
                        %large according to the elements in rdown
                        rsum=sum(RFm,2);
                        rF=find(rsum==max(rsum));
                        lFm=lFm(rF);
                        m=m+1;
                    else
                        rsum=sum(Fm(lFm,:),2); %When Fm reaches Nf, the update ends
                        rF=find(rsum==max(rsum));
                        lFm=lFm(rF(1));
                end
        end
        %%Line replacement
        Arup1=A(rup,:);                                 %Rows to be replaced
        Amax=A(rup+lFm-1,:);
        Arup1(rup)=Arup1(rup+lFm-1);Arup1(rup+lFm-1)=0;         %Arup1 internal replacement
        Amax(rup+lFm-1)=Amax(rup);Amax(rup)=0;                  %Amax internal replacement
        A(rup,:)=Amax;A(rup+lFm-1,:)=Arup1;                     %row Interchange
        A(:,rup)=Amax';A(:,rup+lFm-1)=Arup1';                   %Column Interchange
        r0=r(rup);r(rup)=r(rup+lFm-1);r(rup+lFm-1)=r0;
        %%Reorder within rows
        Ar=A(rup,rup:Nf);                                       %Find the row to adjust
        Fr0=find(Ar==0);
        Fr1=find(Ar==1);
        if length(Fr0)>1
                for i=1:(length(Fr1)-(Fr0(2)-2))                %Start to adjust
                        h0=Fr0(2)-1+i;
                        Ar=A(rup,rup:Nf);
                        Find1=find(Ar((h0+1):length(Ar))==1);
                        h0=rup-1+h0;
                        h1=h0+Find1(1);
                        Artemp0=A(:,h0);                %0 column
                        Artemp0(h0)=Artemp0(h1);Artemp0(h1)=0;
```

```
                Artemp1=A(:,h1);                %1column
                Artemp1(h1)=Artemp1(h0);Artemp1(h0)=0;
                A(:,h0)=Artemp1;                %Adjust 0 column
                A(:,h1)=Artemp0;                %Adjust 1 column
                A(h0,:)=Artemp1';               %Adjust 0 row
                A(h1,:)=Artemp0';               %Adjust 1 row
                r0=r(h0);r(h0)=r(h1);r(h1)=r0;
            end
        end
        rrdown=sum(A(1:rup,:),2)+1;             %Update the rdown before rup
        Rdown=[rrdown;Nf];                      %UpdateRdown
        Rdown=sort(Rdown);                      %Arrange rdown from small to large
        rup=rup+1;                              %Update rup
  end

 %%%Find cliques
 %%The cliques found in this round will be put into the Store
 %%one by one and screened according to the gradual expansion method
 Store=[];
 %Start to search large cliques
 k=2;
 Ar=A(1:k,1:k);
 su=k^2-k;
 while sum(sum(Ar))==su    %If the matrix does not reach the maximum, it will continue to expand until the maximum
       k=k+1;
       Ar=A(1:k,1:k);
       su=k^2-k;
  end
  %After the large clique search, start to find the remaining small connections
  Store(1,:)=r(1:k-1);                          %Put the big clique in the Store
  W=A(1:k-1,k:Nf);                              %W is the matrix to be looked up next to the square matrix
  s=1;
  for i=1:Nf-k+1
        pd=sum(W(:,i));
        if pd>0
            s=s+1;
            nextr=[r(find(W(:,i)>0)),r(i+k-1)];     %nextr stands for small links
            Store(s,1:length(nextr))=nextr;         %Put nextr into the Store
        end
  end
  sizesumStore=size(sumStore);
  sizeStore=size(Store);
  sumStore(sizesumStore(1)+[1:sizeStore(1)],1:sizeStore(2))=Store;     %Put the "Store" in the "sumstore" one by one
end
```

```
%%%Remove duplicate and subset items from sumStore
R=sumStore;                                    %For the convenience of writing, assign sumStore to R
D=[];
rankR=sum(R>0,2);
for i=min(rankR):max(rankR)
        D=[D;R(find(rankR==i),:)];
end
R=D;
RR=[];                                         %Put the identified clique in RR
s1=size(R);
i=0;
while s1(1)>0                                  %As long as there are cliques in R, keep looking
        i=i+1;
        Rr1=R(1,:);
        r0=Rr1(find(Rr1>0));           %r0 is the item greater than 0 in the first row of R
        sR=size(R);
        DR=R(2:sR(1),:);
        sd=1:sR(1)-1;
        for zh=1:length(r0)                            %Find each item in r0 from Dr
                FDR=DR==r0(zh);
                fdr1=find(sum(FDR,2)==0);
                DR(fdr1,:)=[];                         %Remove the cliques in DR that do not contain the items in r0,
                sd(fdr1)=[];                           %and the remaining DR is all cliques that contain (greater than or equal to) r0
        end
        fdr2=find(sum(DR>0,2)==length(r0));    %Remove the clique equal to r0 from DR
        DR(fdr2,:)=[];
        sDR=size(DR);
        %If there is a unique r0, move it to RR. If there is a clique in DR
        %that contains R0, delete the same item in DR,
        %skip R0 and continue to review the next clique
        if sDR(1)==0
                 RR=[RR;R(1,:)];
        end
        R([1,sd(fdr2)+1],:)=[];
        s1=size(R);
end

%In the following, we will screen the paired cliques in RR and fuse them
RD=RR;
[xRD,yRD]=size(RD);
srd=sum(RD>0,2);
ssrd=[];
Sto3=[]; Sto2=[];
```

```
if min(srd)==2                                    %If there are pairs of cliques in RR
        RD2=RD(find(srd==2),1:2);                 %Extract these cliques
        RD100=RD2;
        [x,y]=size(RD2);
        xx=x;
        i=0;                                      %i is the line under review
        while i<x
                i=i+1;
                js=0;
                for j=1:2                                         %Review the elements of each line
                        [x1,y1]=find(RD2(i+1:x,:)==RD2(i,j));
                        if length(x1)>0                   %If there is a duplication with the reviewed element in other lines
                                RD3=RD2(i,:);
                                RD4=[RD2(i,:);RD2(x1+i,:)]; %Put the rows with duplicate elements in RD2 into RD4
                                for s=1:length(x1)
                                        RD3=[RD3,RD2(x1(s)+i,:)]; %Merge the rows with duplicate elements into a vector
                                end
                                RD2(x1+i,:)=[];                           %Remove the lines with duplicate elements in RD2
                                RD3(find(RD3==RD2(i,j)))=[];              %Exclude duplicate elements from a vector
                                z=[]; z1=[];
                                for k=1:length(RD3)
                                        [x2,y2]=find(RD(xx+1:xRD,:)==RD3(k)); %Examine whether different elements are in a clique
                                        z=[z,x2']; z1(k,1:length(x2))=x2';          %Stores the location of elements in RD3 in RD
                                end
                                zmc=[];
                                for m=1:length(z)-1
                                        if sum(z(m+1:length(z))==z(m))==1
                                                zmc=[zmc,z(m)]; %zmc is used to store repeated elements in z
                                        end
                                end
                                if length(zmc)>0
                                        sxz1=[];
                                        for r=1:length(zmc)
                                                [xz1,yz1]=find(z1==zmc(r));
                                                Sto3=[Sto3;RD2(i,j),RD3(xz1),zeros(1,yRD-length([RD2(i,j),RD3(xz1)]))];
                                                sxz1=[sxz1,RD3(xz1)];
                                        end
                                        st=0;
                                        while st<length(sxz1)    %Remove the same elements from sxz1
                                                st=st+1;
                                                sxz1(find(sxz1(st+1:length(sxz1))==sxz1(st)))=[];
                                        end
                                        for s=1:length(RD3)
                                                if sum(sxz1==RD3(s))==0
```

```
                                    Sto3=[Sto3;RD2(i,j),RD3(s),zeros(1,yRD-2)];
                                end
                            end
                        else
                            Sto3=[Sto3;RD4,zeros(length(x1)+1,yRD-2)];
                    end
                else
                    js=js+1;          %Otherwise, count once
            end
            [x,y]=size(RD2);
        end
        if js==2                                %If the same element does not appear in RD2
            Sto2=[Sto2;[RD2(i,:),zeros(1,yRD-2)]]; %It shows that these two elements can form a clique
        end
    end
end
RR=[Sto2;Sto3;RD(sum(RD>0,2)>2,:)];
```
