# Supplementary material for: Simulation of COVID-19 spread through family feast gatherings in a complex network
Source: Epidemiol Infect. 2022 Mar 2;150:e61. doi: 10.1017/S0950268822000292 (PMC8924592; doi:10.1017/S0950268822000292)
Supplement: Supplementary file 1 [file hygsup.zip › S0950268822000292sup002.pdf]

```
clear all
%%RR is the matrix of all cliques
%%H is the adjacency matrix
%%M is the corresponding matrix of family and family members

%%%Start to build family dinner
%%Search the family relationship matrix
Q=[];                                            %It's used to store parents of one's daughter-in-law or son-in-law cliques
R3=RR(find(sum(RR>0,2)==3),:);           %Search cliques with 3 families
[m,n]=size(R3);
for i=1:m
        RD=RR;                                      %Restore RD to RR
        r=RD(i,:);                                   %Temporary storage of cliques to be tested
        RD(i,:)=[];                                  %The original matrix RR removes the rows to be tested
        pd=[];                                       %Used to store families in cliques
        for j=1:3
                [x,y]=find(RD==r(j));
                pd=[pd,x'];
        end
        k=0;                                         %To start a parents of one's daughter-in-law or son-in-law search
        su=0;                                        %Counting
        while k<length(pd)
                k=k+1;
                pd1=pd(k+1:length(pd));
                su=su+sum(pd1==pd(k));
                pd(find(pd1==pd(k))+k)=[];
        end
        if su==2
                Q=[Q;R3(i,:)];
        end
end

%%Set up families in each clique to join the dinner party
RD=RR;
p=0.7;                          %The probability of each family having a feast every day
[m,n]=size(H);                  %m is the number of families
prob=binornd(1,p,1,m);           %If each family have dinner on the same day
RR=RD;
for j=1:length(prob)
         [x,y]=find(RR==j);
         r=round(rand*length(x)+0.5);             %R represents rank
         row1=x(r); col1=y(r);                     %Select the row and column of the clique having a feast
         RR(row1,col1)=j*(prob(j)==1);             %Join the feast (1), not join the feast (0)
         if length(x)>1
```

```
                x(r)=[]; y(r)=[];
                row0=x; col0=y;
                for i=1:length(row0)
                        RR(row0(i),col0(i))=0;
                end
        end
end

%%Get rid of the fact that there are two families in laws having dinner together
%First, find the clique of the in laws
[m,n]=size(Q);
rq=[];                                                  %rq is the serial number of all the families in RD
for i=1:m
        [x,y]=find(RD==Q(i,:));
        rq=[rq,x(1)];
end

%Get rid of in laws with two feasts
RE=RR;
RR=RE;
Rq=RR(rq,:);
frq=find(sum(Rq>0,2)==2);
Rq=Rq(frq,:);                                           %Rq is used to store in laws with two feasts
rq2=rq(frq);                                            %rq2 represents the number of rows in the RR
m_ax=[];
for i=1:length(rq2)
        rRq=Rq(i,:);                                    %Recalculate each row of Rq
        rm=rRq(find(rRq>0));                            %rm stands for the two families in each row of Rq
        for j=1:length(rm)
                [m,n]=find(RD==rm(j));      %m is the family in RM which is in the clique of Rd
                m(find(m==rq2(i)))=[];      %Remove the clique in M
                Rm=RR(m,:);
                m=find(sum(Rm>0,2)>0);
                if length(m)>0
                        maRR=RR(max(m),:);
                        fm=find(maRR==0);
                        maRR(fm(1))=rm(j);
                        m_ax=[m_ax,max(m)];
                end
                RR(rq2(i),find(RR(rq2(i),:)==rm(j)))=0;
        end
end

%%Remove individual meals
```

```
RF=RR;
RR=RF;
fr=find(sum(RR>0,2)==1);                    %Find the row for families that dine alone
R01=RR(fr,:);                               %A matrix of families dining alone
[m,n]=size(R01);
for i=1:m
        r01=R01(i,:);
        sf=r01(find(r01>0));                %Families eating alone
        [x,y]=find(RD==sf);                 %X is the clique in RD of the family dining alone
        x(find(x==fr(i)))=[];
        fa=x(find(sum(RR(x,:)>0,2)>0));     %fa is the optional clique in RR for the family having dinner alone,
                                            %which is the remaining clique after removing the empty clique
        if length(fa)>0
                Rr=RR(fa(round(rand*length(fa)+0.5)),:);    %One clique of RR randomly selected for dinner
                f=find(Rr==0);
                Rr(f(1))=sf;                %Fill in the position of the first 0 in the clique with families waiting for dinner
        end
        RR(fr(i),:)=0;
end

RG=RR;
RR=RG;
Rq=RR(rq,:);
frq=find(sum(Rq>0,2)==2);
Rq=Rq(frq,:);                               %Rq is used to store in laws with two family dinners
rq2=rq(frq);                                %rq2 represents the number of rows in the RR
m_ax=[];
for i=1:length(rq2)
        rRq=Rq(i,:);                        %Recalculate each row of Rq
        rm=rRq(find(rRq>0));                %rm stands for the two families in each row of Rq
        for j=1:length(rm)
                [m,n]=find(RD==rm(j));      %m is the family in rm and the clique in RD
                m(find(m==rq2(i)))=[];      %Remove the clique in m
                Rm=RR(m,:);
                m=find(sum(Rm>0,2)>0);
                if length(m)>0
                        maRR=RR(max(m),:);
                        fm=find(maRR==0);
                        maRR(fm(1))=rm(j);
                        m_ax=[m_ax,max(m)];
                end
                RR(rq2(i),find(RR(rq2(i),:)==rm(j)))=0;
        end
end
```

```
%%Establish the matrix M of family member relationship
[m,n]=size(H);
N=10000;
p=[0.2,0.33,0.28,0.19];
n1=ones(1,N*p(1)); n2=ones(1,N*p(2))*2; n3=ones(1,round(N*p(3)))*3; n4=ones(1,N*p(4))*4;
Nf=[n1,n2,n3,n4];
m2=Nf(randperm(N,m))';
m3=1:sum(m2);
m4=[];
for i=1:m
        m4(i,1:m2(i))=m3(1:m2(i))';
        m3(1:m2(i))=[];
end
M=m4;
```
