# Supplementary material for: Simulation of COVID-19 spread through family feast gatherings in a complex network
Source: Epidemiol Infect. 2022 Mar 2;150:e61. doi: 10.1017/S0950268822000292 (PMC8924592; doi:10.1017/S0950268822000292)
Supplement: Supplementary file 1 [file hygsup.zip › S0950268822000292sup001.pdf]

```
clear all
T=90;                                          %T is the operation deadline
beta=0.8;                                      %Beta indicates the infected probability of an susceptible living or dining with an infected person once
circle=100;                                    %Number of cycles

%%%Establishing H
nfam=1000;                                     %Initial number of families
bn=8;sn=3;
Rf=[];                                         %Store the number of cliques
i=0;
while sum(Rf)~=nfam
            i=i+1;
            Rf=round(rand*(bn-sn+1)+(sn-0.5));        %Equalitily and randomly generate a clique of 3-8 families
            while sum(Rf)<nfam
                Rf=[Rf,round(rand*(bn-sn+1)+(sn-0.5))];
            end
end
E=[];                                          %Used to store all cliques and families
rnfam=1:nfam;
for i=1:length(Rf)
          er=rnfam(1:Rf(i));
          E(i,1:length(er))=er;
          rnfam(1:Rf(i))=[];
end
E2=E;

%%%Find the connection between cliques and store them in matrix F
sRf=round(Rf*0.8);                             %The number of nodes connected by each clique to other cliques
sRf0=sRf;
suRf=zeros(1,length(sRf));                     %Used to store the sum of the degrees of the columns of F
Rf01=zeros(1,length(sRf));                     %Used to determine whether the degree of each column is equal to sRF
F=zeros(length(sRf),length(sRf));              %Used to represent the connection matrix between cliques
k=0;
for i=1:length(Rf)-1
        if Rf01(i)==0
                lRf01=find(Rf01(i+1:length(Rf01))==0); %If the element in Rf01 is equal to 0, the maximum connectivity is not reached
                if sRf(i)<sum(F(i,1:i))+length(lRf01)
                        ran=randperm(length(lRf01),sRf(i)-sum(F(i,1:i))); %Find the order of nodes that can be selected
                        rm=lRf01(ran)+i;                               %Find the node that can be selected
                        suRf(rm)=suRf(rm)+1;                           %Update uRf
                        Rf01=sRf==suRf;                                %UpdateRf01
                        F(i,rm)=1;F(rm,i)=1;                           %UpdateF
                    else
                        Rf01(i)=1;
```

```
                        sRf(i)=sum(F(i,1:i));
                        k=k+1;
                end
        end
end
find(sRf0-sRf~=0);
%%%Establish the final social network, each node represents a family
G=[];                                                   %G is used to store nodes that need to be changed
for i=1:length(Rf)
        G(i,1:sRf(i))=E(i,randperm(Rf(i),sRf(i)));
end

%%Get the E after updating the same node
ran=ones(length(Rf),1);                                 %It is used to store the position that has been replaced
for i=1:length(Rf)-1
        ro=find(F(i,i+1:length(Rf))==1)+i;              %Indicates the line of the family in E that needs to be replaced
        for j=1:sRf(i)-ran(i)+1
                co=find(E(ro(j),:)==G(ro(j),ran(ro(j))));       %Co denotes the column to be replaced in E
                E(ro(j),co)=G(i,ran(i));
                ran(i)=ran(i)+1;
                ran(ro(j))=ran(ro(j))+1;
        end
end

%%Establish the final matrix H
%%Calculating the order of H
eh=[];                          %The matrix E is transformed into row vector and stored in eh
for i=1:length(Rf)
        Er=E(i,:);
        eh=[eh,Er(find(Er>0))];
end
i=1;
while i<length(eh)
        eh(find(eh(i+1:length(eh))==eh(i))+i)=[];      %eh is a vector with non repeating elements
        i=i+1;
end

H=zeros(nfam,nfam);
for i=1:length(eh)
        [m,n]=find(E==eh(i));                   %eh represents the the row of H
        [x,y]=size(E(m,:));
        ec=reshape(E(m,:),1,x*y);
        ec(find(ec==0))=[]; ec(find(ec==eh(i)))=[];     %e0 represents the a column of H
        H(eh(i),ec)=1;H(ec,eh(i))=1;
```

```
end

%%Collecting the relatives by marriage
i=1;
while sum(F(i,i+1:length(Rf)))>0
        rc=find(F(i,i+1:length(Rf))>0)+i;               %fc denotes the clique connected to the i-th clique
        EL=E([i,rc],:);                                 %El denotes all cliques connected to clique i
        su=sum(F(i,i+1:length(Rf))>0);                  %su is the clique coefficient connected to the ith clique
        n=round((su-1)/2);
        ran=round(rand(1,n)*su+0.5);                    %ran represents a row randomly selected from EL
        for j=1:n
                ELr=EL([1,ran(j)+1],:);                 %ELR denotes the clique connected to the first clique in EL
                u=0;                                    %Set the condition for finding two cliques with the same element
                k=0;                                    %The position of the element in ELr1
                while u==0                              %Find the same elements in ELr
                        k=k+1;
                        ELr1=ELr(1,:);ELr2=ELr(2,:);
                        f=find(ELr2==ELr1(k));
                        u=sum(f);
                 end
                 r1=E(i,:); r2=E(rc(ran(j)),:);
                 r1(find(r1==ELr1(k)))=[]; r1=r1(find(r1)>0);
                 r2(find(r2==ELr2(f)))=[]; r2=r2(find(r2)>0);
                 ran1=r1(round(rand*length(r1)+0.5));
                 ran2=r2(round(rand*length(r2)+0.5));
                 H(ran1,ran2)=1;H(ran2,ran1)=1;
        end
        i=i+1;
end
%Remove the unconnected nodes from the network
H(find(sum(H)==0),:)=[];
H(:,find(sum(H)==0))=[];
HD=H;

%%Establish the matrix M of family member relationship
[m,n]=size(H);
N=10000;
p=[0.2,0.33,0.28,0.19];
n1=ones(1,N*p(1)); n2=ones(1,N*p(2))*2; n3=ones(1,round(N*p(3)))*3; n4=ones(1,N*p(4))*4;
Nf=[n1,n2,n3,n4];
m2=Nf(randperm(N,m))';
m3=1:sum(m2);
m4=[];
for i=1:m
```

```
            m4(i,1:m2(i))=m3(1:m2(i))';
            m3(1:m2(i))=[];
end
M=m4;

%%incubation period qfq
nqfq=100000;
OK=makedist('Lognormal','mu',1.357,'sigma',0.764);     %Establishing Lognormal probability density function
qfq=random(OK,1,nqfq);                                  %For storage incubation period
f=find(qfq>14 | qfq<1);
while length(f)~=0
        qf=random(OK,1,length(f));
        qfq(f)=qf;
        f=find(qfq>14 | qfq<1);
end
%%Time from onset to hospitalization
med=10.3+(14.8-10.3)*rand(1,nqfq);                      %med represents ime from onset to hospitalization
B4=[]; B5=[]; B6=[]; B7=[]; cB4=[]; cB5=[]; cB6=[]; cB7=[]; %These empty matrix are used to store different health states individuals
Fsta1=[]; Fsta2=[]; Fsta3=[]; Fsta4=[]; Nfsta1=[]; Nfsta2=[]; Nfsta3=[]; Nfsta4=[];     %It is used to store families and people in different states

for cir=1:circle
            cir
            t=0;                            %T is the time (days)
            nurf=[]; Removef=[];            %nurf is used to store the IDs of moving out families
            H=HD;                           %Removef stores the rows of the families removed from the adjacency matrix H

            %%%Randomly select the patient zero
            [m0,n0]=size(M);
            row=round(rand*m0+0.5);                                 %Randomly select rows
            col=round(rand*length(find(M(row,:)>0))+0.5);           %Randomly select column

            %%%Establish a matrix Fsta to record family health status
            Fsta=zeros(m0,n0+1);      %The first column of the matrix Fsta indicates whether the family is removed from H (0: not removed, 1: removed)
                                      %The following columns indicate the status of family members respectively:
                                      %1. Susceptible period; 2. Incubation period; 3. Infectious period; 4. Treatment period; 5. Death; 6. Rehabilitation
            %%Change all family status in Fsta to 1("susceptible")
            [x,y]=find(M>0);
            for i=1:length(x)
                        Fsta(x(i),y(i)+1)=1;
            end
            fsta=ones(1,m0);
            %%The state of the first source of infection was changed to 3 (infection)
            Fsta(row,col+1)=3;
            [xF,yF]=size(Fsta);
```

```
fsta(row)=2;

B=[];                                        %B represents the matrix for recording cases of infected persons
%%Set the first case, 1. Source of infection; 2. Family number; 3. Personal number; 4. Infection time; 5. Onset time; 6. Hospitalization time;
%%7. Discharge time; 8. (Death as 1, rehabilitation as 0); 9. (1 not found, 2 found)
B(1,1)=0; B(1,2)=row; B(1,3)=M(row,col); B(1,4)=0; B(1,5)=B(1,4)+qfq(round(rand*nqfq+0.5)); B(1,6)=B(1,5)+med(round(rand*nqfq+0.5));
B(1,7)=B(1,6)+12+round(rand*(20-12)+0.5); B(1,8)=binornd(1,0.023); B(1,9)=1;
%The family did not have dinner together during the hospitalization and after death.
Fsta0=[]; Nfsta0=[];        %Fsta0 stores the family status vector fsta, and Nfsta0 stores the number of families in different states

while t<T
        t=t+1;                               %t is the time
        %%%Find the infected person in the incubation period
        f0=find(B(:,4)<=t);         %First find the line in B whose infection time is earlier than t, and f0 represents the line
        Bf0=B(f0,:);                %Bf0 is the intermediate variable
        fi=Bf0(find(Bf0(:,5)>t),3);    %The line with a later onset time than t was found.
                                    %fi indicated that all the infectors entering the incubation period on day t were found
        %%Modify the health status of these infectors in Fsta to the incubation period
        for k=1:length(fi)
                [xfi,yfi]=find(M==fi(k));
                Fsta(xfi,yfi+1)=2;
        end
        %%%Find the infected person during the infectious period
        f1=find(B(:,5)<=t);        %First, find the line in B whose onset time is earlier than t, and f1 represents the line
        Bf1=B(f1,:);               %Bf1 is the intermediate variable
        f2=Bf1(find(Bf1(:,6)>=t),3);
        %If the hospitalization time is later than t, f2 means all the individuals who can attend the dinner party on day t
        %%The status of these infected persons in FSTA was changed to infectious period
        for k=1:length(f2)
                [xf2,yf2]=find(M==f2(k));
                Fsta(xf2,yf2+1)=3;
                fsta(xf2)=2;
        end
        %%%Find the infected person entering the treatment period
        f3=find(B(:,6)<t); %First find the row of B that the hospitalization time is earlier than t, and f3 is the row
        Bf3=B(f3,:);             %Bf3 is the intermediate variable
        f4=Bf3(find(Bf3(:,7)>=t),3);
        %Then, find the row that the discharge time is later than i, f4 means all the individuals entering the treatment period on day t
        removef=[]; comeinf=[]; %remove represents the family that needs to be removed from the adjacency matrix H;
                                 %comeinf represents the family that re enters the adjacency matrix H
        %%Find the families of the infected person entering the treatment period
        for k=1:length(f4)
                [xf4,yf4]=find(M==f4(k));
                Fsta(xf4,yf4+1)=4;
```

```
            %Fsta: 1 susceptible period; 2 latent period; 3 infectious period; 4 treatment period; 5 death; 6 rehabilitation
            if Fsta(xf4,1)==0
                    removef=[removef,xf4];
                    Fsta(xf4,1)=1;
                    fsta(xf4)=3;
            end
end
%%%Find the patients who recovered (died) after discharge
f5=find(B(:,7)<t);
Bf5=B(f5,:);
if length(f5)>0
        fi9=find(Bf5(:,9)==1);
        Bf5=Bf5(fi9,:); %Bf5 refers to the patients who were discharged for rehabilitation (death) for the first time
        [x,y]=size(Bf5);
        if x>0
                f6=Bf5(find(Bf5(:,8)==1),3);        %f6 represents the patient who died
                f7=Bf5(find(Bf5(:,8)==0),3);        %f7 represents the patient who recovered
                f8=[f6;f7];                         %f8 indicates a patient who has recovered and died
                for k=1:length(fi9)
                        B(f5(fi9(k)),9)=2;     %Record the status of the recorded patients as 2
                end
                %%Find the family where the recovered (dead) patient is located
                for k=1:length(f8)
                        [xf8,yf8]=find(M==f8(k));
                        if B(find(B(:,3)==f8(k)),8)==1
                        %If the patient dies, it is only necessary to modify the personal status
                        %in Fsta without adding removef or modifying the family status in Fsta
                                Fsta(xf8,yf8+1)=5;    %Modify personal status
                            else    %If the patient recovers, he needs to attend comef
                                Fsta(xf8,yf8+1)=6;   %Modify personal status
                            If sum(Fsta(xf8,:)==4|Fsta(xf8,:)==5)==0& sum(comeinf==xf8)==0
                              %If there are no patients in the family who are being
                              %treated or who have died, then they can return to H
                                        comeinf=[comeinf,xf8];
                                        Fsta(xf8,1)=0;
                                  %Modify the family status in the first column of Fsta,
                                  %and if the patient recovers, change it to 0
                                        fsta(xf8)=4;
                                end
                        end
                end
        end
end
%%%After upgrading FSTA, find out which infection sources in F2 can enter and
```

```
%%%which ones need to exit (the reason for exit is that their family moved out of H
in=[]; out=[]; outf=[];                %in is the serial number of the entrant; out is the serial number of the outgoing
for k=1:length(f2)
        [xf2,yf2]=find(M==f2(k));
        if Fsta(xf2,1)==0
                in=[in,k];
          else
                out=[out,k];
                if sum(outf==xf2)==0
                        outf=[outf,xf2];       %outf is the family of the outgoing
                end
        end
end
f20=f2(in);
f2out=f2(out);                                   %f2out denotes the infection sources out of the game
f2=f20;                                          %f2 denotes the infection sources into the game
%%Update H, removef means the family that needs to move out of H,
%%comeinf means the family that needs to re-enter H
nurf=[nurf,removef];
Removef=[Removef;H(removef,:)];
H(removef,:)=0; H(:,removef)=0;                  %remove H
if length(comeinf)>0
        for k=1:length(comeinf)                  %re-enter H
                H(comeinf(k),:)=Removef(find(nurf==comeinf(k)),:);
                H(:,comeinf(k))=H(comeinf(k),:)';
                nurf(find(nurf==comeinf(k)))=[];
                Removef(find(nurf==comeinf(k)),:)=[];
        end
end
%Generate RR
%The following procedures refer to supplemental material II
A=H;
sumStore=[];
Sum0=sum(A,2);
Nf=length(Sum0);
greater2=find(Sum0>0);
for bxh=1:length(greater2)
        r=1:Nf;
        A=H;
        r1=greater2(bxh);
        Rdown=[];
        rdown=sum(A(r1,:))+1;
        Rdown=[rdown;Nf];
        Ar1=A(r1,:);
```

```
Ar1(r1)=Ar1(1);Ar1(1)=0;
Ar0=A(1,:);
Ar0(1)=Ar0(r1);Ar0(r1)=0;
A(1,:)=Ar1;A(r1,:)=Ar0;
A(:,1)=Ar1';A(:,r1)=Ar0';
r(1)=r1;r(r1)=1;

Ar=A(1,:);
Fr0=find(Ar==0);
Fr1=find(Ar==1);
if length(Fr0)>1
        for i=1:(length(Fr1)-(Fr0(2)-2))
                h0=Fr0(2)-1+i;
                Ar=A(1,:);
                Find1=find(Ar((h0+1):Nf)==1);
                h1=h0+Find1(1);
                Artemp0=A(:,h0);
                Artemp0(h0)=Artemp0(h1);Artemp0(h1)=0;
                Artemp1=A(:,h1);
                Artemp1(h1)=Artemp1(h0);Artemp1(h0)=0;
                A(:,h0)=Artemp1;
                A(:,h1)=Artemp0;
                A(h0,:)=Artemp1';
                A(h1,:)=Artemp0';
                r0=r(h0);r(h0)=r(h1);r(h1)=r0;
        end
end
rup=2;
while rup<Rdown(1)
        Fm=A(rup:Rdown(1),rup:Nf);
        lFm=1:Rdown(1)-rup+1;
        m=1;
        while length(lFm)>1
                if m<length(Rdown)
                        RFm=Fm(lFm,1:Rdown(m)-rup);
                        rsum=sum(RFm,2);
                        rF=find(rsum==max(rsum));
                        lFm=lFm(rF);
                        m=m+1;
                    else
                        rsum=sum(Fm(lFm,:),2);
                        rF=find(rsum==max(rsum));
                        lFm=lFm(rF(1));
                end
```

```
            end

            Arup1=A(rup,:);
            Amax=A(rup+lFm-1,:);
            Arup1(rup)=Arup1(rup+lFm-1);Arup1(rup+lFm-1)=0;
            Amax(rup+lFm-1)=Amax(rup);Amax(rup)=0;
            A(rup,:)=Amax;A(rup+lFm-1,:)=Arup1;
            A(:,rup)=Amax';A(:,rup+lFm-1)=Arup1';
            r0=r(rup);r(rup)=r(rup+lFm-1);r(rup+lFm-1)=r0;

            Ar=A(rup,rup:Nf);
            Fr0=find(Ar==0);
            Fr1=find(Ar==1);
            if length(Fr0)>1
                    for i=1:(length(Fr1)-(Fr0(2)-2))
                            h0=Fr0(2)-1+i;
                            Ar=A(rup,rup:Nf);
                            Find1=find(Ar((h0+1):length(Ar))==1);
                            h0=rup-1+h0;
                            h1=h0+Find1(1);
                            Artemp0=A(:,h0);
                            Artemp0(h0)=Artemp0(h1);Artemp0(h1)=0;
                            Artemp1=A(:,h1);
                            Artemp1(h1)=Artemp1(h0);Artemp1(h0)=0;
                            A(:,h0)=Artemp1;
                            A(:,h1)=Artemp0;
                            A(h0,:)=Artemp1';
                            A(h1,:)=Artemp0';
                            r0=r(h0);r(h0)=r(h1);r(h1)=r0;
                    end
            end
            rrdown=sum(A(1:rup,:),2)+1;
            Rdown=[rrdown;Nf];
            Rdown=sort(Rdown);
            rup=rup+1;
end

Store=[];
k=2;
Ar=A(1:k,1:k);
su=k^2-k;
while sum(sum(Ar))==su
        k=k+1;
        Ar=A(1:k,1:k);
```

```
                        su=k^2-k;
            end

            Store(1,:)=r(1:k-1);
            W=A(1:k-1,k:Nf);
             s=1;
            for i=1:Nf-k+1
                    pd=sum(W(:,i));
                    if pd>0
                            s=s+1;
                            nextr=[r(find(W(:,i)>0)),r(i+k-1)];
                            Store(s,1:length(nextr))=nextr;
                    end
            end
            sizesumStore=size(sumStore);
            sizeStore=size(Store);
            sumStore(sizesumStore(1)+[1:sizeStore(1)],1:sizeStore(2))=Store;
end

R=sumStore;
D=[];
rankR=sum(R>0,2);
for i=min(rankR):max(rankR)
    D=[D;R(find(rankR==i),:)];
end
R=D;
RR=[];
s1=size(R);
i=0;
while s1(1)>0
        i=i+1;
        Rr1=R(1,:);
        r0=Rr1(find(Rr1>0));
        sR=size(R);
        DR=R(2:sR(1),:);
        sd=1:sR(1)-1;
        for zh=1:length(r0)
                FDR=DR==r0(zh);
                fdr1=find(sum(FDR,2)==0);
                DR(fdr1,:)=[];
                sd(fdr1)=[];
        end
        fdr2=find(sum(DR>0,2)==length(r0));
        DR(fdr2,:)=[];
```

```
            sDR=size(DR);
            if sDR(1)==0
                        RR=[RR;R(1,:)];
            end
            R([1,sd(fdr2)+1],:)=[];
            s1=size(R);
end

RD=RR;
[xRD,yRD]=size(RD);
srd=sum(RD>0,2);
ssrd=[];
Sto3=[]; Sto2=[];
if min(srd)==2
  RD2=RD(find(srd==2),1:2);
  RD100=RD2;
  [x,y]=size(RD2);
  xx=x;
  i=0;
  while i<x
     i=i+1;
     js=0;
     for j=1:2
       [x1,y1]=find(RD2(i+1:x,:)==RD2(i,j));
       if length(x1)>0
          RD3=RD2(i,:);
          RD4=[RD2(i,:);RD2(x1+i,:)];
          for s=1:length(x1)
            RD3=[RD3,RD2(x1(s)+i,:)];
          end
          RD2(x1+i,:)=[];
          RD3(find(RD3==RD2(i,j)))=[];
          z=[]; z1=[];
          for k=1:length(RD3)
            [x2,y2]=find(RD(xx+1:xRD,:)==RD3(k));
            z=[z,x2']; z1(k,1:length(x2))=x2';
          end
          zmc=[];
          for m=1:length(z)-1
            if sum(z(m+1:length(z))==z(m))==1
              zmc=[zmc,z(m)];
            end
          end
          if length(zmc)>0
```

```
                    sxz1=[];
                    for r=1:length(zmc)
                        [xz1,yz1]=find(z1==zmc(r));
                        Sto3=[Sto3;RD2(i,j),RD3(xz1),zeros(1,yRD-length([RD2(i,j),RD3(xz1)]))];
                        sxz1=[sxz1,RD3(xz1)];
                    end
                    st=0;
                    while st<length(sxz1)
                        st=st+1;
                        sxz1(find(sxz1(st+1:length(sxz1))==sxz1(st)))=[];
                    end
                    for s=1:length(RD3)
                       if sum(sxz1==RD3(s))==0
                          Sto3=[Sto3;RD2(i,j),RD3(s),zeros(1,yRD-2)];
                       end
                 end
              else
                   Sto3=[Sto3;RD4,zeros(length(x1)+1,yRD-2)];
        end
      else
        js=js+1;
     end
     [x,y]=size(RD2);
   end
   if js==2
       Sto2=[Sto2;[RD2(i,:),zeros(1,yRD-2)]];
   end
 end
end
RR=[Sto2;Sto3;RD(sum(RD>0,2)>2,:)];                    %The following procedures refer to supplemental material III
     Q=[];
     R3=RR(find(sum(RR>0,2)==3),:);
     [m,n]=size(R3);
     for i=1:m
              RD=RR;
              r=RD(i,:);
              RD(i,:)=[];
              pd=[];
              for j=1:3
                   [x,y]=find(RD==r(j));
                   pd=[pd,x'];
              end
              k=0;
              su=0;
```

```
        while k<length(pd)
                k=k+1;
                pd1=pd(k+1:length(pd));
                su=su+sum(pd1==pd(k));
                pd(find(pd1==pd(k))+k)=[];
        end
        if su==2
                 Q=[Q;R3(i,:)];
        end
end

RD=RR;
[m,n]=size(H);
prob=binornd(1,jp,1,m);
RR=RD;
for j=1:length(prob)
          [x,y]=find(RR==j);
          if x>0
                    r=round(rand*length(x)+0.5);
                    row1=x(r); col1=y(r);
                    RR(row1,col1)=j*(prob(j)==1);
                    if length(x)>1
                              x(r)=[]; y(r)=[];
                              row0=x; col0=y;
                              for i=1:length(row0)
                                        RR(row0(i),col0(i))=0;
                              end
                    end
          end
end

[m,n]=size(Q);
rq=[];
for i=1:m
         [x,y]=find(RD==Q(i,:));
         rq=[rq,x(1)];
end

RE=RR;
RR=RE;
Rq=RR(rq,:);
frq=find(sum(Rq>0,2)==2);
Rq=Rq(frq,:);
rq2=rq(frq);
```

```
m_ax=[];
for i=1:length(rq2)
            rRq=Rq(i,:);
            rm=rRq(find(rRq>0));
            for j=1:length(rm)
                        [m,n]=find(RD==rm(j));
                        m(find(m==rq2(i)))=[];
                        Rm=RR(m,:);
                        m=find(sum(Rm>0,2)>0);
                        if length(m)>0
                                    maRR=RR(max(m),:);
                                    fm=find(maRR==0);
                                    maRR(fm(1))=rm(j);
                                    m_ax=[m_ax,max(m)];
                        end
                        RR(rq2(i),find(RR(rq2(i),:)==rm(j)))=0;
            end
end

RF=RR;
RR=RF;
fr=find(sum(RR>0,2)==1);
R01=RR(fr,:);
[m,n]=size(R01);
for i=1:m
            r01=R01(i,:);
            sf=r01(find(r01>0));
            [x,y]=find(RD==sf);
            x(find(x==fr(i)))=[];
            fa=x(find(sum(RR(x,:)>0,2)>0));
            if length(fa)>0
                        Rr=RR(fa(round(rand*length(fa)+0.5)),:);
                        f=find(Rr==0);
                        Rr(f(1))=sf;
            end
            RR(fr(i),:)=0;
end

RG=RR;
RR=RG;
Rq=RR(rq,:);
frq=find(sum(Rq>0,2)==2);
Rq=Rq(frq,:);
rq2=rq(frq);
```

```
m_ax=[];
for i=1:length(rq2)
        rRq=Rq(i,:);
        rm=rRq(find(rRq>0));
        for j=1:length(rm)
                [m,n]=find(RD==rm(j));
                m(find(m==rq2(i)))=[];
                Rm=RR(m,:);
                m=find(sum(Rm>0,2)>0);
                if length(m)>0
                        maRR=RR(max(m),:);
                        fm=find(maRR==0);
                        maRR(fm(1))=rm(j);
                        m_ax=[m_ax,max(m)];
                end
                RR(rq2(i),find(RR(rq2(i),:)==rm(j)))=0;
        end
end

St=[];    inf=[]; outf2=[];
%St is used to load the cliques formed by all infectious families, inf is used to store the families
%that have formed a bureau (families that can have dinner together),
%and outf2 is used to store the families that have not formed a bureau
if length(f2)>0
        for j=1:length(f2)
                [m,n]=find(M==f2(j));
                [xm,ym]=find(RR==m);            %Verify that the family is in RR
                if xm>0                         %If it's in RR, it means this family can have dinner together
                        inf=[inf,m];
                        Rr=RR(xm,:);
                        Rr0=find(Rr>0);
                        [x,y]=size(St);
                        if x>0 & sum(St(:,Rr0(1))==Rr(Rr0(1)))==0
                          %Since the existing cliques in St may duplicate the newly added cliques,
                          %check whether there is any duplication first
                             St=[St;Rr]; %St refers to the clique that can participate in the dinner party
                          elseif    x==0
                             St=Rr;
                        end
                   elseif sum(outf2==m)==0
                          outf2=[outf2,m];
                          %inf denotes the family in the game, outf2 denotes the family not in the game
                end
        end
```

```
            sumf=[outf,outf2]';
%sumf refers to families that spread within their own homes due to isolation and failure to find a family to eat together
            [xR,yR]=size(RR);
            St0=[];
            if length(sumf)>0
                    St0(:,1)=sumf; St0(:,2:yR)=0;
                    %St0 denotes that all families in sumf are transformed into a matrix connected with St;
            end
            St=[St0;St];      %The matrix St denotes the generalized clique participating in the dinner party
            [xSt,ySt]=size(St);
            for k=1:xSt
                    Stk=St(k,:);
                    Stk=Stk(find(Stk>0));
                    Fs=Fsta(Stk,2:yF);
                    %Fs represents the individual states in the Fsta of all participating families in the state matrix
                    Ms=M(Stk,:);                %Ms refers to the individual corresponding to each family
                    [xFs1,yFs1]=find(Fs==1);
                    [xFs3,yFs3]=find(Fs==3);
                    susp=[]; infp=[];susf=[];
                %Ms refers to all susceptible individuals in St(k,:) and infp refers to all infected individuals in St(k,:);
                    for j=1:length(xFs1)
                         susp=[susp,Ms(xFs1(j),yFs1(j))];
                         [x,y]=find(M==Ms(xFs1(j),yFs1(j)));
                         susf=[susf,x]; %susf carries the families of susceptible members of the k-faction;
                    end
                    for j=1:length(xFs3)
                        infp=[infp,Ms(xFs3(j),yFs3(j))];     %infp completes the loading of all infection sources
                    end
                    p=1-(1-beta)^length(infp);
                    %p is the probability of infection for each susceptible person at each meal
                    fs=find(binornd(1,p,1,length(susp))==1);
                    %intermediate variable, the location of the susceptible
                    infp2=susp(fs);   inff2=susf(fs);
%infp2 is the second generation of infected individuals; inff2 is the family of the second generation of infected individuals
%Matrix B, 1. Source of infection; 2. Family number; 3. Personal number; 4. Time of infection; 5. Time of onset;
%6. Length of hospital stay; 7. Time of discharge; 8. Death as 1, recovery as 0
                 for j=1:length(infp2)
                    [xB,yB]=size(B);
                    B(xB+1,1)=infp(round(rand*length(infp)+0.5)); B(xB+1,2)=inff2(j); B(xB+1,3)=infp2(j);
                    B(xB+1,4)=B(find((B(:,3)==B(xB+1,1))),5)+(t-B(find((B(:,3)==B(xB+1,1))),5))*rand;
                    B(xB+1,5)=B(xB+1,4)+qfq(round(rand*nqfq+0.5));
                     B(xB+1,6)=B(xB+1,5)+med(round(rand*nqfq+0.5));
                    B(xB+1,7)=B(xB+1,6)+12+round(rand*(20-12)+0.5);
                    B(xB+1,8)=binornd(1,0.073); B(xB+1,9)=1;
```

```
                        end
                end
        end
        %1. susceptible family, 2 infected family, 3 treatment family, 4 cure family
        nfsta=sum(M>0,2);
        fsta0=[sum(fsta==1), sum(fsta==2), sum(fsta==3), sum(fsta==4)];
        nfsta0=[sum(nfsta(find(fsta==1))),sum(nfsta(find(fsta==2))),sum(nfsta(find(fsta==3))),sum(nfsta(find(fsta==4)))];
        Fsta0=[Fsta0,fsta0'];
        Nfsta0=[Nfsta0,nfsta0'];
    end
    b4=hist(B(:,4)',[0:T]);          %New infections
    b5=hist(B(:,5)',[0:T]);          %New patients
    b6=hist(B(:,6)',[0:T]);          %New inpatients
    b7=hist(B(:,7)',[0:T]);          %New rehabilitation
    B4=[B4;b4];                      %Summarize the number of new infections in each cycle
    B5=[B5;b5];                      %Summarize the new patients generated by each cycle
    B6=[B6;b6];                      %Summarize the new inpatients generated by each cycle
    B7=[B7;b7];                      %Summarize the number of newly rehabilitated patients in each cycle
    cb4=cumsum(b4);                  %Cumulative infection
    cb5=cumsum(b5);                  %Cumulative patients
    cb6=cumsum(b6);                  %Cumulative inpatients
    cb7=cumsum(b7);                  %Cumulative recovery
    cB4=[cB4;cb4];                   %Summarize cumulative infections per cycle
    cB5=[cB5;cb5];                   %The cumulative number of patients per cycle was summarized
    cB6=[cB6;cb6];                   %The cumulative inpatients generated by each cycle were summarized
    cB7=[cB7;cb7];                   %The cumulative number of patients recovered from each cycle was summarized

    Fsta1=[Fsta1;Fsta0(1,:)];        %Summary of susceptible families
    Fsta2=[Fsta2;Fsta0(2,:)];        %Summary of infected families
    Fsta3=[Fsta3;Fsta0(3,:)];        %Summary treatment family
    Fsta4=[Fsta4;Fsta0(4,:)];        %Family Healing
    Nfsta1=[Nfsta1;Nfsta0(1,:)];     %Total number of susceptible families
    Nfsta2=[Nfsta2;Nfsta0(2,:)];     %Total number of infected families
    Nfsta3=[Nfsta3;Nfsta0(3,:)];     %Total number of families treated
    Nfsta4=[Nfsta4;Nfsta0(4,:)];     %Total number of cured families
end
```
